# Direct and Indirect Parental Influences on Body Image Dissatisfaction in Adult Offspring

**DOI:** 10.1111/eip.70093

**Published:** 2025-10-02

**Authors:** Yasmine Athaide, Stephanie Miles, Eric J. Tan, Andrea Phillipou

**Affiliations:** ^1^ Department of Psychological Sciences Swinburne University of Technology Melbourne Australia; ^2^ Orygen Melbourne Australia; ^3^ Centre for Youth Mental Health, the University of Melbourne Melbourne Australia; ^4^ Memory Aging & Cognition Centre, National University Health System Singapore Singapore; ^5^ Department of Pharmacology Yong Loo Lin School of Medicine, National University of Singapore Singapore Singapore; ^6^ Centre for Mental Health and Brain Sciences, Swinburne University of Technology Melbourne Australia; ^7^ Orygen Specialist Program, Royal Melbourne Hospital Melbourne Australia; ^8^ Department of Mental Health St Vincent's Hospital Melbourne Australia; ^9^ Department of Mental Health Austin Health Melbourne Australia

**Keywords:** body dissatisfaction, body image, eating disorder, offspring, parents

## Abstract

**Introduction:**

Body image dissatisfaction is a risk and maintenance factor for disordered eating. Parents may contribute to offspring body image dissatisfaction through weight‐related criticism (direct influence), as well as expressed parental body image dissatisfaction and dieting (indirect influence). This study retrospectively investigated the contribution of parental influence towards adult offspring's body image dissatisfaction.

**Methods:**

One hundred and fifty six participants from a general community sample completed a survey recording their experience of direct and indirect parental influence throughout adolescence, in addition to their current body image satisfaction.

**Results:**

While controlling for peer and media influences, hierarchical linear regression determined that parental influence was a significant contributor to offspring body image dissatisfaction (*p* < 0.001), and this effect was driven by direct influence (*p* < 0.001). Indirect parental influence was non‐significant (*p* = 0.899).

**Conclusions:**

The observed strength of direct influence supports the need for parents to reinforce positive weight‐related behaviours at home as a method of reducing and managing body image dissatisfaction levels in their offspring.

## 1 Introduction

Body image dissatisfaction has been recognised as a robust predictor of eating disorder symptomatology in both clinical and non‐clinical populations (Phelps et al. 1999; Polivy and Herman 2002; Rosewall et al. 2018). One of the most significant outcomes of body image dissatisfaction is its contribution towards the development of various eating disorders (Stice and Shaw 2002), with some suggesting it is the single strongest predictor of eating disorder symptoms among women (Polivy and Herman 2002). As body image concerns typically emerge during adolescence (Voelker et al. 2015), successfully managing body dissatisfaction may thus prevent the onset of eating disorder development and mitigate symptom severity, suggesting a need to better characterise contributors to elevated body image dissatisfaction.

One of the earliest forms of socialisation is via parent–child interactions (McCabe and Ricciardelli 2003); therefore, family and parental dynamics have been extensively considered regarding the development and maintenance of poor body image (Keel et al. 1997). Parents have often been considered to contribute to significant levels of body image dissatisfaction (Rodgers et al. 2009), with weight discussion and teasing estimated to occur overtly and covertly in approximately two‐thirds of households (Berge et al. 2015). Their influence is recognised within the tripartite influence model for eating psychopathology, which identifies three forms of sociocultural influence upon disordered eating and body image: media, peers, and parents (Thompson et al. 1999). This model proposes that these influences motivate internalisation of the thin ideal and social comparison of appearance, resultantly encouraging body image dissatisfaction and thus contributing to eating disorder symptomatology. Although some research has observed that these pathways mediate the relationship between parental influence and offspring body image dissatisfaction (Keery et al. 2004; Yamamiya et al. 2008), contradictory findings have also been reported (Rodgers et al. 2011; Shroff and Thompson 2006). Internalisation and social comparison may thus explain the mechanisms of peer and media influence; however, this trajectory may be less applicable to parental influence.

Alternative theories have suggested the role of parents may be based more strongly on explicit attitudes and behaviours. Two modes of parental influence have been proposed to explain the association between offspring body image dissatisfaction and parental contribution: direct and indirect parental influence. Direct parental influence occurs via parental teasing or criticism towards their offspring's weight, eating behaviours, or body shape. Direct influence also describes when parents encourage offspring to lose or control their weight (Abraczinskas et al. 2012; Fulkerson et al. 2002; Vincent and McCabe 2000). Indirect parental influence describes when an offspring's body image and eating behaviours reflect parents' own expressions of body image dissatisfaction, and through displays of negative parental eating behaviours, which may then be mimicked by offspring (Abraczinskas et al. 2012). Thus, the ‘thin ideal’ is indirectly reinforced by parents and observable diet techniques are deemed acceptable to manage weight concerns (Wertheim et al. 1999).

Direct parental influence has been consistently implicated for its ability to predict offspring body image dissatisfaction. Research has revealed that parental weight loss encouragement, in addition to parental teasing, is positively associated with body image dissatisfaction in adolescent females (Ata et al. 2007; Hanna and Bond 2006; Keery et al. 2005; Wertheim et al. 2002). Over a 1‐year period, Helfert and Warschburger (2011) observed parental weight loss encouragement to be predictive of male and female adolescent weight concern. This observation has been mirrored in young adult samples (Schwartz et al. 1999; Vázquez and Santoncini 2019). Contrasting findings have been observed; however, this may be because weight‐related comments can be perceived as expressions of maternal caring, rather than negatively (Marquez and Benitez 2021).

Research investigating the role of indirect parental influence has displayed less consistency. Maternal and paternal body image dissatisfaction has been associated with female offspring body image dissatisfaction (Handford et al. 2018; Keery et al. 2006; Kichler and Crowther 2001; Lowes and Tiggemann 2003; Perez La Mar et al. 2018), yet this relationship has not been mirrored between mothers and their sons (Elfhag and Linné 2005; Fulkerson et al. 2002). Furthermore, maternal and paternal dieting has also been associated with disordered eating in daughters (Handford et al. 2018; Neumark‐Sztainer et al. 2010; Vincent and McCabe 2000; Wertheim et al. 1999). Parental dieting also contributed to offspring body image dissatisfaction in these studies, but to a lesser extent (Neumark‐Sztainer et al. 2010; Vincent and McCabe 2000). Despite these observations, longitudinal research highlights an evident disparity in this area. Longitudinally, research has observed both significant (Klein et al. 2017; van den Berg et al. 2010) and non‐significant (Byely et al. 2000; Paxton et al. 2006) findings between indirect parental influences and adolescent offspring body image dissatisfaction. However, when these indirect influences are more extreme, such as in cases where parents have eating disorder diagnoses, the likelihood of disordered eating patterns emerges in their children (Bould et al. 2015).

When both forms of influence are considered together, inconsistent findings are observed regarding the strength of each influence (Abraczinskas et al. 2012; Bardone‐Cone et al. 2011; Wertheim et al. 1999). The evidence base suggests that direct parental influence may be more prominent than indirect messages that may or may not be negatively perceived by offspring. Encouragement to diet and direct weight commentary from parents to their offspring have displayed prominent salience over parental diet modelling in relation to offspring body dissatisfaction (Smolak et al. 1999; Wertheim et al. 2002). Despite these observations in cross‐sectional research, longitudinal data suggest that the two forms of influence may be more equally weighted. Some research has observed that parental encouragement to lose weight and indirect dieting discussions equally contributed to adolescent body image dissatisfaction in females over a 4‐year period (Blodgett‐Salafia and Gondoli 2011). This finding was endorsed by 20‐year longitudinal research conducted with adults, whereby maternal dieting and paternal weight‐related comments significantly predicted drive for thinness in women (Klein et al. 2017). As such, there is a clear discrepancy in the strength of each influence that prompts further exploration.

As body image concerns usually emerge around adolescence (Helfert and Warschburger 2011), there is a relative paucity of body image research involving adult‐aged offspring. When adults are the focus of this research area, retrospective measures are often employed. High levels of perceived direct parental influences during childhood and adolescence have been shown to be strong predictors of adult offspring body image dissatisfaction (Kluck 2010). Additionally, research in this area often detects the significance of both influences, yet greater contribution towards offspring body image dissatisfaction is typically via direct parental influence (Bardone‐Cone et al. 2011). Other retrospective literature has also suggested that direct parental influences are more related to offspring eating disorder symptoms, while indirect influences may display a greater effect on body image dissatisfaction (Abraczinskas et al. 2012); however, this has yet to be replicated.

Building upon prior research, the aim of the current study was to investigate how direct and indirect parental influence contributes to offspring body image dissatisfaction in a community‐based adult sample, while controlling for peer and media influences. It was hypothesised that whilst controlling for peer and media influences, (1) direct and indirect parental influence would have a significant combined association with offspring body image dissatisfaction, and that (2) this association would be stronger for direct parental influence than for indirect parental influence.

## 2 Method

### 2.1 Participants

Eligibility criteria required all participants to be 18 years or older, and currently residing in Australia. A total of 270 individuals were recruited via convenience sampling. Eighty six cases were deleted listwise due to missing data on the survey, and an additional seven participants were removed due to failing two or more of three attention checks throughout the survey. The final sample comprised of 177 individuals, including 156 females (88.1%) and 21 males (11.9%). Ages ranged from 18 to 82 years old (M = 29.92, SD = 10.71). Most of the sample was born in Australia (80.2%) and identified as Caucasian (78.5%), with the remainder of the sample identifying as Asian (13.6%), Aboriginal or Torres Strait Islander (1.7%), African (0.6%), or other (6.2%). The average length of education for the final sample was 16.6 years of formal education (SD = 3.7).

### 2.2 Procedure

After gaining ethics approval from the Swinburne University Human Research Ethics Committee (SUHREC), the study was advertised through the Swinburne University research experience program, via online platforms such as the Butterfly Foundation, via social media posts (Facebook and Instagram), and through participant registries held at Swinburne University. Eligible participants were directed to an online Qualtrics survey which they completed at their own convenience. Participants reviewed the consent information statement and had the opportunity to clarify outstanding questions via email before participating. After completing the consent form online, participants completed a range of questionnaires for a larger study; only the measures relevant to the current study are described here. Participants who completed the survey through the research experience program received course credit, whilst all other participants were placed into a draw to win one of eight $25 vouchers. All data were collected between June and July 2022.

### 2.3 Materials and Measures

#### 2.3.1 Parental Influence

Parental influence was assessed using the parental influence questionnaire (PIQ; Abraczinskas et al. 2012). This 28‐item scale was used to assess the degree to which an individual's body image and eating behaviours are influenced by their parents. The PIQ contains two subscales measuring direct parental influence consisting of 18 items (e.g., ‘my parents made negative comments about my physical appearance’), and indirect influence consisting of 10 items (e.g., ‘my parents complained about their weight’). The scale was slightly modified to ask participants to rate items in relation to the interactions they had with their parents as a child or adolescent. Responses were rated on a 5‐point Likert scale ranging from 1 = *strongly disagree* to 5 = *strongly agree*. Items on each subscale were averaged to produce two overall scores depicting experienced level of direct and indirect parental influence. Thus, scores ranged from one to five, whereby higher scores indicated greater experience of each respective parental influence. Research has observed excellent and good reliability for the direct (*α* = 0.91) and indirect (*α* = 0.84) parental influence subscales, respectively (Abraczinskas et al. 2012). In the current study, excellent reliability was observed for both the direct (*α* = 0.95) and indirect (*α* = 0.90) subscales.

#### 2.3.2 Body Image Dissatisfaction

The eating disorder examination questionnaire (EDE‐Q; Fairburn and Beglin 2008) is a 28‐item scale used to assess body image and disordered eating symptomatology. The EDE‐Q is comprised of four subscales: restraint, eating concern, shape concern, and weight concern. For this study, only the shape and weight concern subscales (13 items in total) were used to reflect body image dissatisfaction. The shape concern subscale contains eight items (e.g., ‘have you had a definite desire to have a totally flat stomach?’), and the weight concern subscale contains five items (e.g., ‘have you ever had a strong desire to lose weight?’). Participants were asked to respond on a 7‐point Likert scale indicating the number of days in the past month they practiced or experienced various features associated with poor body image (0 = *no days*, to 6 = *every day*). Scores on the shape and weight subscales were combined and averaged to reflect current overall body image dissatisfaction levels. Scores ranged from zero to six, whereby higher scores indicated higher levels of current body image dissatisfaction. Past research has reported excellent and good reliability for the shape (*α* = 0.90) and weight (*α* = 0.85) concern subscales, respectively (Rose et al. 2013). In the present study, excellent and good reliability was observed for the shape (*α* = 0.94) and weight (*α* = 0.88) concern subscales.

#### 2.3.3 Demographic and Control Variables

Demographic data, including age and sex, were collected. Other relevant demographic information was also obtained, including current living situation and mental health history. More specifically, participants were asked to self‐identify whether they had ever had a diagnosis of an eating disorder. Peer and media influences were also recorded as per the tripartite influence model to control for these factors in the statistical models. Peer and media influence was assessed using the tripartite influence scale (TIS; Keery et al. 2004). This 43‐item scale assesses the influence of sociocultural factors, specifically media, peer, and parental influence upon an individual's body image and behaviours of disordered eating. The peer influence subscale consists of 13 items (e.g., ‘how often do your friends and classmates comment on each other's weight?’), while the media influence subscale consists of 10 items (e.g., ‘the magazines I read and the TV shows I watch emphasise dieting to lose weight’). Participants were asked to indicate the regularity of such experiences and behaviours occurring on a 5‐point Likert scale (1 = *always*, 5 = *never*). Items on each subscale were averaged to produce two overall scores depicting level of peer and media influence. Scores ranged from one to five, whereby lower scores indicated greater experience of these influences upon body image. This scale has been reported to have good reliability for the peer subscale (*α* = 0.89) and acceptable reliability for the media subscale (*α* = 0.77; You et al. 2018). In the current study, reliability analyses revealed excellent reliability for the peer subscale (*α* = 0.94) and good reliability for the media subscale (*α* = 0.84).

### 2.4 Data Preparation and Statistical Analysis

All statistical analyses were conducted using IBM Statistical Package for Social Sciences 28.0.1. Hierarchical linear regression was used to examine whether direct and indirect parental influence contribute to offspring body image dissatisfaction. Demographic variables (age and sex) were entered into Block 1, followed by other influence variables (peer and media influence) in Block 2. Lastly, the direct and indirect parental influence variables were entered into Block 3 together to allow for direct comparisons to occur between them.

The collected data was screened for outliers and compliance with assumptions of hierarchical regression analysis. Independence of errors was examined using the Durbin–Watson statistic and was deemed acceptable (DW = 1.80). Homoscedasticity was examined via scatterplot, and normality was assessed via P–P plot of the regression standardised residuals. Additionally, multicollinearity was examined via assessment of variance inflation factors (VIF; O'Brien 2007). All assumptions were met, and no outliers were observed.

## 3 Results

Demographic information and descriptive statistics for the variables included in the regression analysis are reported in Table 1.

**TABLE 1:** Sample characteristics.

	%	M (SD)
Past or current self‐reported eating disorder diagnosis	45.2%	—
Living arrangements		
Living alone	19.2%	—
Living with unrelated adults	10.2%	—
Couple living together with no children	19.2%	—
Couple living together with children	13.6%	—
Single parent living with children	4.5%	—
Single and living with family	24.3%	—
Couple living with family	7.9%	—
Other	1.1%	—
Parental influence questionnaire		
Direct parental influence	—	2.7 (1.1)
Indirect parental influence	—	3.3 (1.0)
Tripartite influence scale		
Peer influence	—	3.1 (0.9)
Media Influence	—	3.1 (0.8)
Body image dissatisfaction	—	3.1 (1.8)

*Note: N* = 177; body image dissatisfaction was calculated by averaging the shape and weight concern subscales of the EDE‐Q.

Results of the associated correlation analyses and hierarchical linear regression are reported in Tables 2 and 3.

**TABLE 2:** Pearson's correlations.

Variable	1	2	3	4	5	6	7
1. Body image dissatisfaction	—						
2. Age	−0.08	—					
3. Sex^a^	0.30***	−0.05	—				
4. Peer influence	−0.39***	−0.15*	−0.10	—			
5. Media influence	−0.31***	0.004	−0.21**	0.49***	—		
6. Direct parental influence	0.45***	−0.04	0.16*	−0.32***	−0.14*	—	
7. Indirect parental influence	0.35***	−0.23***	0.25***	−0.30***	−0.20**	0.59***	—

*Note: N* = 177; sex was coded as males = 1 and females = 2; *p < 0.05, **p < 0.01, ***p < 0.001.

^a^ Eta value.

**TABLE 3:** Hierarchical linear regression model of body image dissatisfaction.

	Variable	Standardised, β	SE	95% CI		*p*
				LL	UL	
Block 1	Age	−0.089	0.011	−0.038	0.007	0.181
	Sex	0.199	0.368	0.388	1.843	0.003
Block 2	Peer influence	−0.226	0.163	−0.798	−0.153	0.004
	Media influence	−0.113	0.172	−0.604	0.074	0.124
Block 3	Direct influence	0.330	0.133	0.289	0.814	< 0.001
	Indirect influence	−0.011	0.149	−0.314	0.276	0.899

*Note: N* = 177.

Abbreviations: CI, confidence interval; LL, lower limit; UL, upper limit.

As observed in Table 2, correlational analyses indicated that being female was associated with higher levels of body image dissatisfaction. A significant negative correlation was identified between body image dissatisfaction and both peer and media influences, indicating that greater body image dissatisfaction was associated with greater peer and media influence. Additionally, body image dissatisfaction was positively correlated with both direct and indirect parental influence.

As observed in Table 3, age and sex accounted for 9.4% of variance explained (*R* ^2^ = 0.09, *F* = 9.04, *p* < 0.001), and the overall model was significant, *F*(2,174) = 9.04, *p* < 0.001. When peer and media influence were entered into the model, a further 14.8% of variance was explained (Δ*R* ^2^ = 0.15, *R* ^2^ = 0.24, *F* = 16.81, *p* < 0.001). The overall model also remained significant, *F*(4,172) = 13.74, *p* < 0.001. When direct and indirect parental influence were included, a further 9.1% of variance was explained (Δ*R* ^2^ = 0.09, *R* ^2^ = 0.33, *F* = 11.66, *p* < 0.001), and the overall model remained significant, *F*(6,170) = 14.19, *p* < 0.001. The analysis of beta values revealed that direct parental influence made a significant contribution towards offspring body image dissatisfaction (*p* < 0.001); however, the contribution of indirect parental influence was non‐significant (*p* = 0.899). All VIFs were within acceptable range (< 2) and indicated an absence of overlapping effects (O'Brien 2007).

The exclusion of participants with a history of eating disorders did not meaningfully alter the direction or statistical significance of the results. As such, these individuals were retained in the final analysis to preserve statistical power.

## 4 Discussion

The current study examined the contribution of parental influence during adolescence towards adult offspring body image dissatisfaction, while specifically investigating the discrete effect of direct (e.g., criticism/teasing of offspring's weight) and indirect (e.g., negative parental eating behaviours) parental influence. The first hypothesis, that direct and indirect parental influences would significantly contribute to offspring body image dissatisfaction, was partially supported for direct parental influence only. Furthermore, the second hypothesis was supported, whereby direct parental influence produced a greater contribution towards offspring body image dissatisfaction than indirect parental influence.

The effect of direct parental influence on offspring body image dissatisfaction has been well supported in past literature. The current findings thus reflect a highly consistent body of research displaying the contribution of parental weight‐related teasing and encouragement to diet upon offspring body image dissatisfaction (Ata et al. 2007; Hanna and Bond 2006; Keery et al. 2005; Vincent and McCabe 2000; Wertheim et al. 2002). Such a robust finding proposes that negative verbal commentary and encouragement to lose weight as expressed by parents may be instrumental in the development and later maintenance of body image dissatisfaction in their adult offspring. Through utilisation of an adult population, this observation particularly highlights the pervasive nature of direct parental influence.

The present study also supports past research depicting the importance of direct influence above indirect influence on body image dissatisfaction development and related outcomes in both non‐retrospective (Smolak et al. 1999; Wertheim et al. 1999) and retrospective (Bardone‐Cone et al. 2011; Schwartz et al. 1999) research. Although both types of research observe significance for both direct and indirect parental influences, direct influences appear consistently more salient upon comparison (Bardone‐Cone et al. 2011; Wertheim et al. 1999). Although not investigated in the present study, there may be potential differentiation within the behaviours involved in direct influences. Despite parental weight‐related commentary and encouragement to diet both being categorised as direct influence, each behaviour may contribute distinguishable levels of impact towards offspring body image dissatisfaction. Differentiating the strength of each specific behaviour will provide clarity regarding the relative strength of explicit attitudes and behaviours upon offspring body image.

While the results of the current study support much of the existing literature in the field, the process and trajectory of parental influence may be different when considering longitudinal research. Longitudinally, the research literature is highly variable, with both significant (Blodgett‐Salafia and Gondoli 2011; Bould et al. 2015; Klein et al. 2017; van den Berg et al. 2010) and non‐significant (Byely et al. 2000; Paxton et al. 2006) findings being observed for both forms of influence. As such, the cross‐sectional nature of the current study may be less sensitive to capturing the long‐term, cumulative impacts of more subtle and habitual parental behaviours as observed in longitudinal research (Klein et al. 2017). Additionally, non‐significant findings are often observed when parental influences are considered among other predictors such as peer influence (Paxton et al. 2006). Greater longitudinal research is required to clarify this relationship over time, and in the presence of other predictors.

The current study contributes to an inconsistent body of research surrounding indirect parental influence. In relation to expressed maternal and paternal body image dissatisfaction, both significant (Keel et al. 1997; Keery et al. 2006; Kichler and Crowther 2001) and non‐significant (Elfhag and Linné 2005; Fulkerson et al. 2002) contributions have been observed towards offspring dissatisfaction levels. There are various potential explanations as to why indirect parental influences were not associated with body image dissatisfaction in the present study. Firstly, some research has observed a significant effect of indirect influence only under high levels of direct parental influence, thus proposing a potential moderation effect (Kichler and Crowther 2001). As the level of direct influence was, on average, lower than indirect influence, a moderation effect may have occurred. Furthermore, outcomes of indirect parental influence often differ depending upon who is reporting parental behaviours (Rodgers and Chabrol 2009). Parental body image dissatisfaction and dieting reported by parents are often not associated with negative offspring outcomes, yet this relationship becomes significant when these behaviours are perceived and reported by offspring (Haines et al. 2008; Keery et al. 2006). Although relying on offspring perception of parental behaviours and attitudes may be considered limiting, such perceptions may yet have discrete salience above parental reports.

Another possible explanation for the lack of an observed effect of indirect parental influence is that such influences may be more pronounced in early childhood and diminish over time. Longitudinal research by Wang et al. (2019) suggests that body dissatisfaction becomes relatively stable by mid‐adolescence, indicating that earlier childhood may represent a more sensitive period for such influences. Resultantly, relying on retrospective reports from adult participants may attenuate these early effects, leading to an underestimation of indirect parental influences in adulthood. Future longitudinal research beginning in early childhood may better capture the precise developmental timeline. Additionally, although the measure utilised for indirect parental influence in the current study is considered reliable, it may lack sensitivity as a retrospective assessment tool.

Importantly, the current study did not differentiate behaviours within direct and indirect influence, despite each set of behaviours often being related to differing outcomes in past research. For example, parental dieting appears to align closer with disordered eating in offspring (Neumark‐Sztainer et al. 2010; Vincent and McCabe 2000; Wertheim et al. 1999), whilst expressed parental body image dissatisfaction appears to relate more to offspring body image dissatisfaction (Keery et al. 2006; Lowes and Tiggemann 2003). As these behaviours were not discriminated, and disordered eating was not analysed, it is unknown if one form of behaviour produced the non‐significant results observed. Future research may benefit from distinguishing between these two forms of behaviour to better assess their relative impact on specific outcomes in offspring.

It is well understood that body image dissatisfaction is a risk factor for the development and maintenance of eating disorder symptomatology (Polivy and Herman 2002; Rosewall et al. 2018). The results of the current study thus highlight the pervasiveness of negative parental comments and weight loss encouragement during an offspring's formative years, thus potentially contributing to their body image concerns as adults.

### 4.1 Strengths, Limitations, and Future Directions

As adolescents are typically of greater research interest, exploration involving an adult sample is less common. As such, utilising a community‐based adult sample advances current understanding of how parental attitudes and behaviours contribute to and maintain offspring body image concerns. Additionally, peer and media influences were controlled for, thus allowing parental influence alone to be focused upon. Much of the existing literature that distinguishes between direct and indirect parental influences has not accounted for these broader social contexts. The present study demonstrates that, even when these factors are controlled for, parents continue to make a significant and unique contribution to offspring body image dissatisfaction. Additionally, the present study utilised multi‐item measures with established psychometric properties to assess parental influences, thereby enhancing the interpretability of the findings. This approach allows for a more comprehensive assessment compared to prior studies that have often relied on single‐item indicators.

However, these findings must be considered in light of some limitations. This study is cross‐sectional in nature, whereby causality cannot be inferred. Additionally, retrospective assessment of parental influence may be biased by the current parent–child relationship quality and perceptions, particularly since a large proportion of participants resided with their family at the time of the study. Such biases may have differentially affected reports of direct and indirect parental influences. Given the emotionally charged nature of direct parental influences, such comments may be more vividly recalled—either accurately or distorted by current relationship dynamics (Puhl and Himmelstein 2018). In contrast, indirect parental influences may be more subtle and habitual (Dickens and Ogden 2014), making them more susceptible to reinterpretation (e.g., ‘They were just eating healthy’), or even complete omission when recalled retrospectively. Although retrospective self‐reports are subject to potential recall biases, they may still offer valid insights into parental behaviours. Research has suggested that perceived parental influences can be more predictive of psychological outcomes than objective measures (Cooley et al. 2008). Notably, offspring reports of parental attitudes and behaviours were confirmed by spousal reports in research conducted by Keel et al. (1997), supporting the relevance and validity of offspring recall. To minimise these limitations, cross‐sectional research that utilises the same measures of direct and indirect parental influences across age groups, or preferably longitudinal research assessing the same participants over time (including observational and ecological momentary assessment methods), should be adopted.

Additionally, a large portion of participants within the community‐based sample reported past and/or current eating disorders, which may suggest that body image dissatisfaction on average was heightened in the sample. Future studies may benefit from determining the influence of parents upon individuals with formal diagnoses (not merely self‐report) of past or current experience of an eating disorder and thus, those of clinical interest.

Due to the limited number of male participants, potential gender differences could not be evaluated. While this study did not aim to analyse gender differences, the body image dissatisfaction scale used in the present study may not have accurately captured common body image concerns experienced by males, including muscularity concerns (Jones and Crawford 2005). Since the EDE‐Q measures concern surrounding the thin ideal, application to males or those with muscularity‐related concerns is thus limited. Utilising a measure that differentiates between sources for body dissatisfaction will be pivotal in clarifying gender differences in body image concerns. In a similar way, given the gender disproportion—specifically the underrepresentation of male and gender‐diverse populations—the study has limited generalisability to the broader adult population.

Additionally, the mean age of the sample in the current study was 30 years. While not currently assessed, different generations across the lifespan may have variable experiences. Future research may distinguish between different developmental periods regarding parental influence and body image dissatisfaction to determine generational differences.

Finally, the media influence measure used in the present study primarily captured traditional media sources such as magazines and television. As social media has become an increasingly dominant force in shaping body image, future research would benefit from incorporating new measures that reflect a changing context of social influences (i.e., the consumption of popular and emerging types of social media), particularly for younger generations.

### 4.2 Clinical Implications

Although not assessed in the present study, much past research has clustered the effect of body image dissatisfaction with dieting and excessive exercise, ultimately fostering disordered eating symptomatology (Polivy and Herman 2002; Rosewall et al. 2018). As such, the ways in which parents promote the maintenance of poor body image in their offspring can be critical in the later development of harmful outcomes. Such knowledge may inform intervention and educational programs that can provide parental guidance for constructing a household that cultivates positive weight‐related behaviours and attitudes among offspring. Thus, potentially, body image concerns may be minimised and prevent the development of subsequent eating disorder psychopathology.

## 5 Conclusions

This study endeavoured to examine how past direct and indirect parental influences contribute to current adult offspring body image dissatisfaction in a non‐clinical sample. Importantly, by controlling for peer and media influences, the findings of this study highlight the unique and meaningful contribution of parents as a significant source of influence upon body image dissatisfaction. The results suggest that parents may influence offspring body dissatisfaction directly through weight‐related criticism and encouragement to diet. While such commentary may not be intentionally harmful and perhaps may be delivered to promote a healthy lifestyle, such influences may contribute to poor body image in adult offspring. Thus, greater research elucidating the role of parental influence on body image dissatisfaction in adult offspring will aid our understanding of how these factors contribute to body image dissatisfaction and how we could provide better education to parents about their potential influence on their children. While the current findings support existing longitudinal research in this area, prospective, longitudinal research that measures both parental behaviours and offspring body image dissatisfaction over time is needed to understand how these explicit parental modes of influence change throughout the offspring's life.

## Ethics Statement

Ethics approval was acquired through the Swinburne University Human Research Ethics Committee (SUHREC).

## Consent

All participants provided informed consent to participate in this study.

## Conflicts of Interest

The authors declare no conflicts of interest.

## Data Availability

The data that support the findings of this study are available from the corresponding author upon reasonable request.

## References

[eip70093-bib-0001] Abraczinskas, M. , B. Fisak , and R. D. Barnes . 2012. “The Relation Between Parental Influence, Body Image, and Eating Behaviors in a Nonclinical Female Sample.” Body Image 9: 93–100. 10.1016/j.bodyim.2011.10.005. 22104125

[eip70093-bib-0002] Ata, R. N. , A. B. Ludden , and M. M. Lally . 2007. “The Effects of Gender and Family, Friend, and Media Influences on Eating Behaviors and Body Image During Adolescence.” Journal of Youth and Adolescence 36, no. 8: 1024–1037. 10.1007/s10964-006-9159-x.

[eip70093-bib-0003] Bardone‐Cone, A. M. , M. B. Harney , and L. Sayen . 2011. “Perceptions of Parental Attitudes Toward Body and Eating: Associations With Body Image Among Black and White College Women.” Body Image 8, no. 2: 186–189. 10.1016/j.bodyim.2010.12.001. 21354878

[eip70093-bib-0004] Berge, J. M. , A. Trofholz , S. Fong , L. Blue , and D. Neumark‐Sztainer . 2015. “A Qualitative Analysis of Parents' Perceptions of Weight Talk and Weight Teasing in the Home Environments of Diverse Low‐Income Children.” Body Image 15: 8–15. 10.1016/j.bodyim.2015.04.006. 25978273 PMC4643419

[eip70093-bib-0005] Blodgett‐Salafia, E. H. , and D. M. Gondoli . 2011. “A 4‐Year Longitudinal Investigation of the Processes by Which Parents and Peers Influence the Development of Early Adolescent Girls' Bulimic Symptoms.” Journal of Early Adolescence 31, no. 3: 390–414. 10.1177/0272431610366248.

[eip70093-bib-0006] Bould, H. , U. Sovio , I. Koupil , et al. 2015. “Do Eating Disorders in Parents Predict Eating Disorders in Children? Evidence From a Swedish Cohort.” Acta Psychiatrica Scandinavica 132, no. 1: 51–59. 10.1111/acps.12389. 25572654

[eip70093-bib-0007] Byely, L. , A. B. Archibald , J. Graber , and J. Brooks‐Gunn . 2000. “A Prospective Study of Familial and Social Influences on Girls' Body Image and Dieting.” International Journal of Eating Disorders 28, no. 2: 155–164. 10.1002/1098-108x(200009)28:2<155::aid-eat4>3.0.co;2-k. 10897077

[eip70093-bib-0009] Cooley, E. , T. Toray , M. C. Wang , and N. N. Valdez . 2008. “Maternal Effects on Daughters' Eating Pathology and Body Image.” Eating Behaviors 9: 52–61. 10.1016/j.eatbeh.2007.03.001. 18167323

[eip70093-bib-0010] Dickens, E. , and J. Ogden . 2014. “The Role of Parental Control and Modelling in Predicting a Child's Diet and Relationship With Food After They Leave Home. A Prospective Study.” Appetite 76: 23–29. 10.1016/j.appet.2014.01.013. 24480669

[eip70093-bib-0011] Elfhag, K. , and Y. Linné . 2005. “Gender Differences in Associations of Eating Pathology Between Mothers and Their Adolescent Offspring.” Obesity Research 13, no. 6: 1070–1076. 10.1038/oby.2005.125. 15976150

[eip70093-bib-0012] Fairburn, C. G. , and S. J. Beglin . 2008. “Eating Disorder Examination Questionnaire (EDE‐Q 6.0).” In Cognitive Behavior Therapy and Eating Disorders, edited by C. G. Fairburn , 309–313. Guilford Press.

[eip70093-bib-0014] Fulkerson, J. A. , M. T. McGuire , D. Neumark‐Sztainer , M. Story , S. A. French , and C. L. Perry . 2002. “Weight‐Related Attitudes and Behaviors of Adolescent Boys and Girls Who Are Encouraged to Diet by Their Mothers.” International Journal of Obesity 26, no. 12: 1579–1587. 10.1038/sj.ijo.0802157. 12461674

[eip70093-bib-0015] Haines, J. , D. Neumark‐Sztainer , P. Hannan , and R. Robinson‐O'Brien . 2008. “Child Versus Parent Report of Parental Influences on Children's Weight‐Related Attitudes and Behaviors.” Journal of Pediatric Psychology 33, no. 7: 783–788. 10.1093/jpepsy/jsn016. 18304997 PMC2734118

[eip70093-bib-0016] Handford, C. M. , R. M. Rapee , and J. Fardouly . 2018. “The Influence of Maternal Modeling on Body Image Concerns and Eating Disturbances in Preadolescent Girls.” Behaviour Research and Therapy 100: 17–23. 10.1016/j.brat.2017.11.001. 29128584

[eip70093-bib-0017] Hanna, A. C. , and M. J. Bond . 2006. “Relationships Between Family Conflict, Perceived Maternal Verbal Messages, and Daughters' Disturbed Eating Symptomatology.” Appetite 47, no. 2: 205–211. 10.1016/j.appet.2006.02.013. 16701919

[eip70093-bib-0018] Helfert, S. , and P. Warschburger . 2011. “A Prospective Study on the Impact of Peer and Parental Pressure on Body Dissatisfaction in Adolescent Girls and Boys.” Body Image 8, no. 2: 101–109. 10.1016/j.bodyim.2011.01.004. 21354379

[eip70093-bib-0019] Jones, D. C. , and J. K. Crawford . 2005. “Adolescent Boys and Body Image: Weight and Muscularity Concerns as Dual Pathways to Body Dissatisfaction.” Journal of Youth and Adolescence 34, no. 6: 629–636. 10.1007/s10964-005-8951-3.

[eip70093-bib-0020] Keel, P. K. , T. F. Heatherton , J. L. Harnden , and C. D. Hornig . 1997. “Mothers, Fathers, and Daughters: Dieting and Disordered Eating.” Eating Disorders 5, no. 3: 216–228. 10.1080/10640269708249227.

[eip70093-bib-0021] Keery, H. , K. Boutelle , P. van den Berg , and J. K. Thompson . 2005. “The Impact of Appearance‐ Related Teasing by Family Members.” Journal of Adolescent Health 37, no. 2: 120–127. 10.1016/j.jadohealth.2004.08.015. 16026721

[eip70093-bib-0022] Keery, H. , M. E. Eisenberg , K. Boutelle , D. Neumark‐Sztainer , and M. Story . 2006. “Relationships Between Maternal and Adolescent Weight‐Related Behaviors and Concerns: The Role of Perception.” Journal of Psychosomatic Research 61, no. 1: 105–111. 10.1016/j.jpsychores.2006.01.011. 16813852

[eip70093-bib-0023] Keery, H. , P. van den Berg , and J. K. Thompson . 2004. “An Evaluation of the Tripartite Influence Model of Body Dissatisfaction and Eating Disturbance With Adolescent Girls.” Body Image 1, no. 3: 237–251. 10.1016/j.bodyim.2004.03.001. 18089156

[eip70093-bib-0024] Kichler, J. C. , and J. H. Crowther . 2001. “The Effects of Maternal Modeling and Negative Familial Communication on Women's Eating Attitudes and Body Image.” Behavior Therapy 32, no. 3: 443–457. 10.1016/S0005-7894(01)80030-7.

[eip70093-bib-0025] Klein, K. M. , T. A. Brown , G. A. Kennedy , and P. K. Keel . 2017. “Examination of Parental Dieting and Comments as Risk Factors for Increased Drive for Thinness in Men and Women at 20‐Year Follow‐Up.” International Journal of Eating Disorders 50, no. 5: 490–497. 10.1002/eat.22599. 27526950

[eip70093-bib-0026] Kluck, A. S. 2010. “Family Influence on Disordered Eating: The Role of Body Image Dissatisfaction.” Body Image 7: 8–14. 10.1016/j.bodyim.2009.09.009. 19945366

[eip70093-bib-0027] Lowes, J. , and M. Tiggemann . 2003. “Body Dissatisfaction, Dieting Awareness and the Impact of Parental Influence in Young Children.” British Journal of Health Psychology 8: 135–147. 10.1348/135910703321649123. 12804329

[eip70093-bib-0028] Marquez, B. , and T. Benitez . 2021. “Individual and Family Factors in Disordered Eating Patterns of Mexican‐American Women.” American Journal of Health Behavior 45, no. 6: 1050–1058. 10.5993/AJHB.45.6.9. 34969416 PMC10005836

[eip70093-bib-0029] McCabe, M. P. , and L. A. Ricciardelli . 2003. “Sociocultural Influences on Body Image and Body Changes Among Adolescent Boys and Girls.” Journal of Social Psychology 143: 5–26. 10.1080/00224540309598428. 12617344

[eip70093-bib-0030] Neumark‐Sztainer, D. , K. W. Bauer , S. Friend , P. J. Hannan , M. Story , and J. M. Berge . 2010. “Family Weight Talk and Dieting: How Much Do They Matter for Body Dissatisfaction and Disordered Eating Behaviors in Adolescent Girls?” Journal of Adolescent Health 47, no. 3: 270–276. 10.1016/j.jadohealth.2010.02.001. PMC2921129 20708566

[eip70093-bib-0031] O'Brien, R. M. 2007. “A Caution Regarding Rules of Thumb for Variance Inflation Factors.” Quality and Quantity: International Journal of Methodology 41, no. 5: 673–690. 10.1007/s11135-006-9018-6.

[eip70093-bib-0032] Paxton, S. J. , M. E. Eisenberg , and D. Neumark‐Sztainer . 2006. “Prospective Predictors of Body Dissatisfaction in Adolescent Girls and Boys: A Five‐Year Longitudinal Study.” Developmental Psychology 42, no. 5: 888–899. 10.1037/0012-1649.42.5.888. 16953694

[eip70093-bib-0033] Perez La Mar, M. , A. M. Kroon Van Diest , H. Smith , et al. 2018. “Body Dissatisfaction and Its Correlates in 5‐ To 7‐Year‐Old Girls: A Social Learning Experiment.” Journal of Clinical Child and Adolescent Psychology 47, no. 5: 757–769. 10.1080/15374416.2016.1157758. 27254542

[eip70093-bib-0034] Phelps, L. , L. S. Johnston , and K. Augustyniak . 1999. “Prevention of Eating Disorders: Identification of Predictor Variables.” Eating Disorders: The Journal of Treatment & Prevention 7, no. 2: 99–108. 10.1080/10640269908251189.

[eip70093-bib-0035] Polivy, J. , and C. P. Herman . 2002. “Causes of Eating Disorders.” Annual Review of Psychology 53: 187–213. 10.1146/annurev.psych.53.100901.135103. 11752484

[eip70093-bib-0036] Puhl, R. M. , and M. S. Himmelstein . 2018. “A Word to the Wise: Adolescent Reactions to Parental Communication About Weight.” Childhood Obesity (Print) 14, no. 5: 291–301. 10.1089/chi.2018.0047. 29975556

[eip70093-bib-0037] Rodgers, R. , and H. Chabrol . 2009. “Parental Attitudes, Body Image Disturbance and Disordered Eating Amongst Adolescents and Young Adults: A Review.” European Eating Disorders Review: The Journal of the Eating Disorders Association 17, no. 2: 137–151. 10.1002/erv.907. 19130467

[eip70093-bib-0038] Rodgers, R. , H. Chabrol , and S. J. Paxton . 2011. “An Exploration of the Tripartite Influence Model of Body Dissatisfaction and Disordered Eating Among Australian and French College Women.” Body Image 8, no. 3: 208–215. 10.1016/j.bodyim.2011.04.009. 21664887

[eip70093-bib-0039] Rodgers, R. F. , S. J. Paxton , and H. Chabrol . 2009. “Effects of Parental Comments on Body Dissatisfaction and Eating Disturbance in Young Adults: A Sociocultural Model.” Body Image 6, no. 3: 171–177. 10.1016/j.bodyim.2009.04.004. 19464242

[eip70093-bib-0040] Rose, J. S. , A. Vaewsorn , F. Rosselli‐Navarra , G. T. Wilson , and R. S. Weissman . 2013. “Test‐Retest Reliability of the Eating Disorder Examination‐Questionnaire (EDE‐Q) in a College Sample.” Journal of Eating Disorders 1: 42. 10.1186/2050-2974-1-42. 24999420 PMC4081765

[eip70093-bib-0041] Rosewall, J. K. , D. H. Gleaves , and J. D. Latner . 2018. “An Examination of Risk Factors That Moderate the Body Dissatisfaction‐Eating Pathology Relationship Among New Zealand Adolescent Girls.” Journal of Eating Disorders 6: 38. 10.1186/s40337-018-0225-z. 30473790 PMC6240946

[eip70093-bib-0042] Schwartz, D. J. , V. Phares , S. Tantleff‐Dunn , and J. K. Thompson . 1999. “Body Image, Psychological Functioning, and Parental Feedback Regarding Physical Appearance.” International Journal of Eating Disorders 25, no. 3: 339–343. 10192000 10.1002/(sici)1098-108x(199904)25:3<339::aid-eat13>3.0.co;2-v

[eip70093-bib-0055] Shroff, H. , and J. K. Thompson . 2006. “The Tripartite Influence Model of Body Image and Eating Disturbance: A Replication With Adolescent Girls.” Body image 3, no. 1: 17–23. 18089205 10.1016/j.bodyim.2005.10.004

[eip70093-bib-0043] Smolak, L. , M. P. Levine , and F. Schermer . 1999. “Parental Input and Weight Concerns Among Elementary School Children.” International Journal of Eating Disorders 25, no. 3: 263–271. 10.1002/(sici)1098-108x(199904)25:3<263::aid-eat3>3.0.co;2-v. 10191990

[eip70093-bib-0044] Stice, E. , and H. E. Shaw . 2002. “Role of Body Dissatisfaction in the Onset and Maintenance of Eating Pathology: A Synthesis of Research Findings.” Journal of Psychosomatic Research 53, no. 5: 985–993. 10.1016/s0022-3999(02)00488-9. 12445588

[eip70093-bib-0045] Thompson, J. K. , L. J. Heinberg , M. Altabe , et al. 1999. “Future Directions: Integrative Theories, Multidimensional Assessments, and Multicomponent Interventions.” In Exacting Beauty: Theory, Assessment, and Treatment of Body Image Disturbance, edited by J. K. Thompson , L. J. Heinberg , M. Altabe , and S. Tantleff‐Dunn , 311–332. American Psychological Association. 10.1037/10312-011.

[eip70093-bib-0046] van den Berg, P. A. , H. Keery , M. Eisenberg , and D. Neumark‐Sztainer . 2010. “Maternal and Adolescent Report of Mothers' Weight‐Related Concerns and Behaviors: Longitudinal Associations With Adolescent Body Dissatisfaction and Weight Control Practices.” Journal of Pediatric Psychology 35, no. 10: 1093–1102. 10.1093/jpepsy/jsq042. 20498008 PMC2980944

[eip70093-bib-0047] Vázquez, C. D. D. L. , and C. U. Santoncini . 2019. “Parental Negative Weight/Shape Comments and Their Association With Disordered Eating Behaviours: A Systematic Review.” Mexican Journal of Eating Disorders 10: 134–147. 10.22201/fesi.20071523e.2019.1.572.

[eip70093-bib-0048] Vincent, M. A. , and M. P. McCabe . 2000. “Gender Differences Among Adolescents in Family, and Peer Influences on Body Dissatisfaction, Weight Loss, and Binge Eating Behaviors.” Journal of Youth and Adolescence 29, no. 2: 205–221. 10.1023/A:1005156616173.

[eip70093-bib-0049] Voelker, D. K. , J. J. Reel , and C. Greenleaf . 2015. “Weight Status and Body Image Perceptions in Adolescents: Current Perspectives.” Adolescent Health, Medicine and Therapeutics 6: 149–158. 10.2147/AHMT.S68344. 26347007 PMC4554432

[eip70093-bib-0050] Wang, S. B. , A. F. Haynos , M. M. Wall , C. Chen , M. E. Eisenberg , and D. Neumark‐Sztainer . 2019. “Fifteen‐Year Prevalence, Trajectories, and Predictors of Body Dissatisfaction From Adolescence to Middle Adulthood.” Clinical Psychological Science: A Journal of the Association for Psychological Science 7, no. 6: 1403–1415. 10.1177/2167702619859331. 32864198 PMC7451946

[eip70093-bib-0051] Wertheim, E. H. , G. Martin , M. Prior , A. Sanson , and D. Smart . 2002. “Parent Influences in the Transmission of Eating and Weight Related Values and Behaviors.” Eating Disorders 10, no. 4: 321–334. 10.1080/10640260214507. 16864275

[eip70093-bib-0052] Wertheim, E. H. , V. Mee , and S. J. Paxton . 1999. “Relationships Among Adolescent Girls' Eating Behaviors and Their Parents' Weight‐Related Attitudes and Behaviors.” Sex Roles: A Journal of Research 41, no. 3–4: 169–187. 10.1023/A:1018850111450.

[eip70093-bib-0053] Yamamiya, Y. , H. Shroff , and J. K. Thompson . 2008. “The Tripartite Influence Model of Body Image and Eating Disturbance: A Replication With a Japanese Sample.” International Journal of Eating Disorders 41: 88–91. 10.1002/eat.20444. 17968899

[eip70093-bib-0054] You, S. , K. Shin , and E. K. Kim . 2018. “The Effects of Sociocultural Pressures and Exercise Frequency on the Body Esteem of Adolescent Girls in Korea.” Journal of Child and Family Studies 27: 26–33. 10.1007/s10826-017-0866-6.

